# Case report: Epidermolysis bullosa acquisita following dipeptidyl peptidase-4 inhibitor therapy and complicated by immune thrombocytopenic purpura

**DOI:** 10.3389/fimmu.2025.1724412

**Published:** 2026-01-05

**Authors:** Hirofumi Kawamoto, Natsuko Sasaki, Yukimi Ueda, Norito Ishii, Yu Sawada

**Affiliations:** 1 Department of Dermatology, University of Occupational and Environmental Health, Kitakyushu, Japan; 2 Department of Dermatology, Kurume University Hospital, Kurume, Japan

**Keywords:** epidermolysis bullosa acquisita, dipeptidyl peptidase-4 inhibitor, immune thrombocytopenic purpura, linagliptin, case report

## Abstract

Epidermolysis bullosa acquisita (EBA) is a rare autoimmune blistering disease caused by autoantibodies to type VII collagen. While dipeptidyl peptidase-4 (DPP-4) inhibitors are established triggers for bullous pemphigoid (BP), their association with EBA has not been reported. A 68-year-old Japanese man with type 2 diabetes and chronic renal dysfunction, treated with linagliptin, developed widespread tense blisters with mucosal involvement. Histopathology and immunological studies confirmed EBA. Despite corticosteroids, cyclosporine, pulse therapy, and intravenous immunoglobulin, the disease remained refractory. Eighty days after onset, he developed pneumonia, renal failure requiring dialysis, and severe thrombocytopenia. After exclusion of other causes, immune thrombocytopenic purpura (ITP) was clinically diagnosed. Although treatment yielded transient platelet recovery, the patient ultimately died. This is the first reported case of EBA following DPP-4 inhibitor therapy complicated by ITP. It highlights the therapeutic challenges of EBA and the potential for systemic autoimmune manifestations beyond the skin in patients receiving DPP-4 inhibitors.

## Introduction

Epidermolysis bullosa acquisita (EBA) is a rare, chronic autoimmune subepidermal blistering disease caused by autoantibodies against type VII collagen, a major component of anchoring fibrils at the dermal–epidermal junction (1, 2). Clinically, EBA often mimics bullous pemphigoid (BP) with widespread tense bullae and subepidermal clefting, but unlike BP, it typically follows a refractory course with frequent relapses and poor response to conventional immunosuppressive therapies (3).

Dipeptidyl peptidase-4 (DPP-4), also known as CD26, is a ubiquitously expressed serine protease involved in glucose homeostasis, immune regulation, and T-cell activation through the cleavage of incretins and various chemokines. DPP-4 inhibitors, widely prescribed for type 2 diabetes mellitus, have been strongly associated with the onset of BP (4). Patients with DPP-4 inhibitor–associated BP are generally elderly, often exhibit non-inflammatory phenotypes, and frequently improve after drug discontinuation (5). However, to date, no case of EBA triggered by DPP-4 inhibitors has been documented.

EBA has also been reported in association with autoimmune and systemic conditions such as thyroiditis, rheumatoid arthritis, hepatitis C infection, and diabetes mellitus (6), suggesting that it may arise in the context of broader immune dysregulation. Nonetheless, the coexistence of EBA with immune thrombocytopenic purpura (ITP), another antibody-mediated autoimmune disease, has not been described.

Herein, we report a challenging case of DPP-4 inhibitor–associated EBA complicated by ITP that ultimately resulted in a fatal outcome. This case underscores the therapeutic difficulties of EBA and highlights the potential for systemic autoimmune complications beyond the skin.

## Case presentation

**Figure 1:**
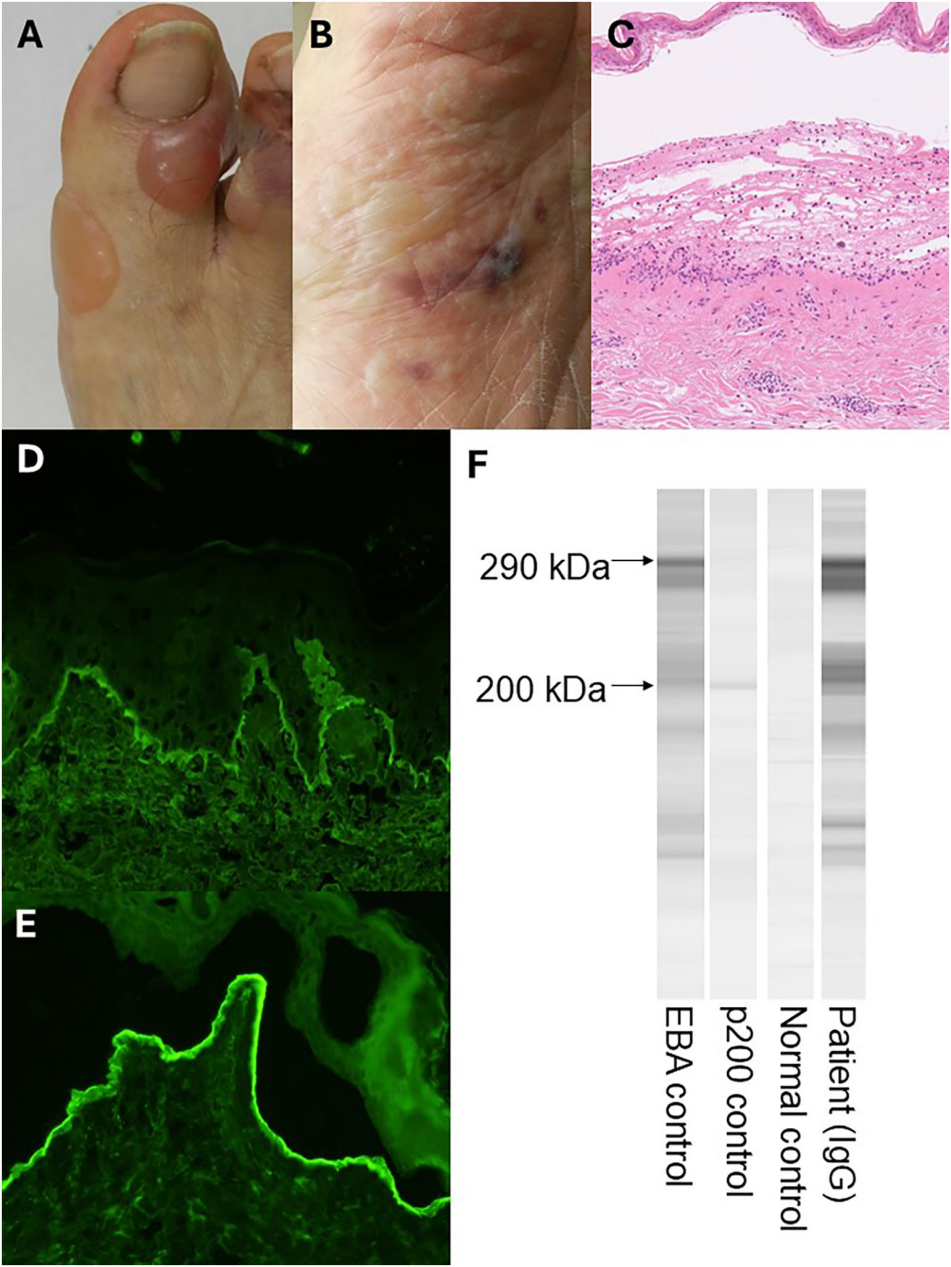
Clinical, histopathologic, and immunologic findings of the patient. (**A, B**) Tense bullae on the right first toe and plantar surface. **(C)** Skin biopsy specimen from the abdomen showing subepidermal blister formation with lymphocytic infiltration in the dermis. **(D)** Direct immunofluorescence (DIF). IgG deposition was observed in the basement membrane zone. **(E)** Indirect immunofluorescence (IIF) using 1M NaCl-split skin demonstrating IgG deposition on the dermal side. **(F)** Immunoblotting. Immunoblotting revealed a positive band corresponding to the 290-kDa type VII collagen antigen.

A 68-year-old Japanese man with long-standing type 2 diabetes mellitus had been receiving linagliptin for the past 10 years, with stable glycemic control. The patient had long-standing chronic kidney disease, with serum creatinine gradually rising into the 5–6 mg/dL range prior to the onset of EBA, later progressing to dialysis-dependent renal failure during hospitalization. Two weeks prior to referral, he developed widespread blisters, initially suspected to represent dipeptidyl peptidase-4 (DPP-4) inhibitor–associated bullous pemphigoid (BP), given the well-established link between DPP-4 inhibitors and autoimmune blistering disorders at that time. On physical examination, multiple tense blisters were observed on the right first toe and plantar surface (**Figures 1A, B**). A skin biopsy from an erythematous plaque on the abdomen revealed subepidermal blistering with lymphocytic infiltration in the dermis and an absence of eosinophils (**Figure 1C**). These histological findings were atypical for BP, prompting consideration of alternative diagnoses, including epidermolysis bullosa acquisita (EBA). Direct immunofluorescence (DIF) of perilesional skin demonstrated linear IgG deposition along the basement membrane zone (**Figure 1D**). Indirect immunofluorescence (IIF) on 1 M NaCl–split skin was performed, revealing IgG deposition on the dermal side (**Figure 1E**), a finding characteristic of EBA. Immunoblotting using normal human dermal extract revealed IgG autoantibodies against the 290-kDa type VII collagen, while antibodies to BP180, p200, and laminin-332 were not detected (**Figure 1F**).

Upon referral, linagliptin was immediately discontinued; however, despite drug withdrawal, the blistering progressively worsened over the following week. Immediately after linagliptin cessation, initial therapy with oral prednisolone (20 mg/day) and cyclosporine (100 mg/day) was started, but the blisters remained uncontrolled and soon flared. One week later, methylprednisolone pulse therapy (1,000 mg/day for 3 days) was administered. Intravenous immunoglobulin (IVIg) was then given at 400 mg/kg/day for 5 consecutive days; however, new lesions continued to appear. Plasmapheresis was avoided in this case because of the patient’s active infection and high risk of procedure-related complications. In addition, rituximab and biologics were not pursued because they are not approved for EBA under the Japanese national insurance system.

Eighty days after disease onset, the patient developed pneumonia with hypoxemia, and his renal function deteriorated, requiring dialysis. Hemodynamic instability prompted admission to the intensive care unit (ICU). During the ICU stay, his platelet count fell sharply to 1.0 × 10³/µl. Coagulation studies did not meet the criteria for disseminated intravascular coagulation (DIC), and there was no evidence of thrombotic microangiopathy. Drug-induced thrombocytopenia was also considered, given his exposure to multiple antibiotics and antifungal agents. In accordance with current ITP diagnostic guidelines (7), we systematically excluded secondary causes of thrombocytopenia. There were no features of thrombotic microangiopathy such as schistocytes or laboratory evidence of hemolysis. Anti–platelet antibody testing was negative; however, given the limited sensitivity of this assay, a negative result does not exclude ITP. Bone marrow examination could not be performed due to the patient’s profound thrombocytopenia, hemodynamic instability, and sepsis. The transient platelet improvement following intravenous immunoglobulin further supported an immune-mediated thrombocytopenia consistent with ITP. Subsequently, intravenous immunoglobulin and escalation of corticosteroids were administered, resulting in a favorable hematologic response with recovery of platelet counts.

## Discussion

DPP-4 inhibitors are well established as a trigger for BP, with multiple pharmacovigilance studies and case series confirming this association (5, 8–11). The underlying mechanism involves DPP-4/CD26–mediated immune dysregulation. DPP-4/CD26 acts as a co-stimulatory molecule on T cells and regulates cytokine secretion. Its inhibition alters immune homeostasis, disturbing the balance among Th1, Th2, Th17, and Tregs (12). Experimental studies show that DPP-4 is preferentially expressed on Th1 and Th17 cells (13, 14). Since pharmacological inhibition downregulates Th1 responses, this immune shift may explain why BP, a Th2-driven disease, is the most common autoimmune blistering disorder associated with DPP-4 inhibitors.

In contrast, EBA is driven by Th1/Th17 pathways that promote neutrophil-mediated dermal–epidermal junction damage (3, 14). Recent experimental data demonstrate that blocking IFN-γ signaling ameliorates disease activity in EBA models (13), highlighting a central role for Th1 cytokines in EBA pathogenesis. One possible explanation for the paradoxical development of EBA in the setting of DPP-4 inhibition is that DPP-4 blockade may not fully suppress Th1/Th17 activation in certain inflammatory states, allowing residual or compensatory pathways to sustain a Th1/Th17-dominant environment despite pharmacologic inhibition.

Another possible mechanism is epitope spreading. Drug-induced immune dysregulation may initially trigger autoimmunity against accessible antigens such as BP180. However, persistent inflammation and tissue damage can expose deeper dermal antigens, leading to a secondary immune response against type VII collagen. Because type VII collagen is normally sequestered within anchoring fibrils (3), its recognition under chronic inflammatory conditions may account for the rarity of EBA compared with BP and highlights the potential of DPP-4 inhibitors to broaden the autoimmune target spectrum.

A notable clinical feature in our patient was that withdrawal of linagliptin did not lead to improvement of EBA. In most cases of DPP-4 inhibitor–associated BP, skin lesions improve within weeks to months after discontinuation (15). In contrast, our patient remained refractory despite drug withdrawal and intensive immunosuppressive therapies. This observation indicates that once an autoimmune response against type VII collagen is established, it may become self-sustaining and independent of the initial pharmacologic trigger. Clinically, this underscores the need for dermatologists to distinguish between BP and EBA in patients on DPP-4 inhibitors, as therapeutic responses and prognosis may differ substantially.

A progressive decline in renal function was evident in our patient, with serum creatinine gradually rising into the 5–6 mg/dL range, consistent with advanced chronic kidney disease. Chronic renal dysfunction is known to impair immune tolerance (16). Such long-standing immune dysregulation may have lowered the threshold for autoimmune activation, offering a plausible explanation for the delayed onset of EBA despite long-term stable linagliptin use. Advanced renal impairment may also have contributed to the refractory disease course and the lack of improvement after discontinuation of the DPP-4 inhibitor.

A further striking aspect of this case was the complication of ITP, which has not been reported in association with DPP-4 inhibitor–related autoimmune blistering diseases. ITP is an acquired autoimmune disorder that may occur in the context of systemic autoimmune conditions such as systemic lupus erythematosus, where secondary ITP develops in 20–30% of patients (12). Its coexistence with EBA is extremely rare. Although HLA associations have been reported separately in each disorder (17, 18), no common genetic basis has yet been identified. Instead, both diseases share immunological features characterized by Th1/Th17 polarization and impaired regulatory T-cell function. Elevated levels of IL-17 and IFN-γ have been described in ITP (19). This overlap raises the possibility that DPP-4 inhibition may lower the threshold for systemic autoimmune activation, thereby predisposing susceptible individuals to simultaneous cutaneous and hematologic autoimmunity.

Emerging evidence implies that DPP-4/CD26 is broadly involved in immune regulation across multiple organ systems, functioning as a co-stimulatory molecule on activated T cells and as a regulator of cytokine secretion and chemokine cleavage. In predisposed individuals, pharmacologic inhibition of DPP-4 may disrupt immune tolerance in more than one tissue compartment. The concurrent development of EBA mediated by a Th1/Th17-driven cutaneous autoimmune disease and immune-mediated thrombocytopenia in our patient raises the possibility that DPP-4 inhibition can precipitate a “multi-organ autoimmunity syndrome.” This concept is supported by reports describing DPP-4 inhibition–associated thyroiditis, arthritis, and other immune-mediated conditions, suggesting that the loss of CD26-dependent regulatory control may lower the threshold for autoimmune activation in genetically or immunologically susceptible hosts. Thus, rather than provoking a single disease, DPP-4 blockade may create a permissive immunologic environment in which multiple autoimmune pathways are concurrently unmasked.

## Conclusion

Taken together, our case demonstrates that DPP-4 inhibitors, while most often associated with BP, can rarely precipitate EBA, and that this may be accompanied by additional systemic autoimmune manifestations such as ITP. To our knowledge, this is the first report of EBA complicated by ITP in the context of DPP-4 inhibitor therapy. These findings underscore the importance of maintaining vigilance for atypical and refractory blistering diseases in patients receiving DPP-4 inhibitors and of considering broader systemic autoimmune complications beyond the skin.

## Data Availability

The original contributions presented in the study are included in the article/supplementary material. Further inquiries can be directed to the corresponding author.
